# Lightweight Underwater Sonar Object Detection via RGB-Guided Heterogeneous Distillation

**DOI:** 10.3390/s26144340

**Published:** 2026-07-08

**Authors:** Qianqian Qiao, Jia Liu, Feng Liu, Chengpeng Hao, Tongwei Ren

**Affiliations:** 1 State Key Laboratory for Novel Software Technology, Nanjing University, Nanjing 210093, China; qiaoqq@smail.nju.edu.cn; 2 Laboratory of Autonomous Underwater Vehicles, Institute of Acoustics, Chinese Academy of Sciences, Beijing 100190, China; liujia@mail.ioa.ac.cn (J.L.); haochengp@mail.ioa.ac.cn (C.H.); 3 National Innovation Institute of Defense Technology, Academy of Military Sciences, Beijing 100097, China; liufeng_cv@126.com

**Keywords:** underwater object detection, cross-modal heterogeneous distillation, lightweight student model, CA-enhanced backbone, sonar sensing

## Abstract

Underwater object detection is a fundamental task in underwater sensing and is generally approached using either optical or sonar sensors. Although optical imaging provides rich semantic information, it is highly susceptible to water turbidity and illumination variations. By contrast, sonar imaging can effectively overcome visibility limitations, yet it suffers from severe speckle noise and blurred object contours. Moreover, resource-limited platforms impose strict demands on model lightweightness and real-time performance. To this end, this paper proposes a novel cross-modal heterogeneous distillation method (CMHD) to balance detection accuracy and computational complexity. CMHD performs cross-modal knowledge transfer by leveraging the rich semantics of RGB images to enhance sonar feature representation, compensating for the information deficiency of the sonar modality. Meanwhile, a heterogeneous distillation scheme compresses the detection capability of a high-capacity teacher YOLOX-M into a lightweight student YOLOX-S-Ghost, enabling strong feature extraction under a highly compact model. To mitigate the modality gap and geometric inconsistency between RGB and sonar modalities, we design a branch-aware heterogeneous distillation strategy. To improve detection accuracy and reduce model parameters, the student network incorporates Coordinate Attention (CA) in its backbone and adopts a lightweight neck design. Experiments on the UXO^†^ dataset demonstrate that CMHD achieves 79.6% mAP and 82.6% mAR, significantly outperforming the compared representative methods and serving as an accurate, efficient, and lightweight solution for underwater sonar object detection.

## 1. Introduction

Underwater object detection is a key component of sensor-based underwater perception and is of great significance for marine security, ecological monitoring, and seabed engineering. Representative tasks include unexploded ordnance clearance [1,2,3], rare aquatic organism monitoring [4,5], and seabed infrastructure inspection [6]. Compared with optical vision sensors, sonar sensors are less sensitive to water turbidity and illumination variations, and are therefore widely used in underwater perception. However, due to the acoustic imaging mechanism and complex sea conditions, sonar images often exhibit low spatial resolution, strong speckle noise, and blurred target boundaries. These characteristics make object detection challenging, especially in scenarios with weak target echoes and strong background clutter. This intrinsic lack of information makes it difficult for unimodal sonar detectors to capture sufficient semantically discriminative cues.

In recent years, deep learning-based detectors, particularly the YOLO series [7,8,9,10], have significantly advanced underwater perception research. Compared with traditional hand-crafted feature methods such as threshold segmentation and template matching [11,12], CNN-based methods achieve stronger representational capacity through automatic feature extraction [13,14,15]. Models such as Faster R-CNN [16] and the YOLO series [17,18,19] have been applied to underwater sonar detection tasks. Among them, YOLOX [9], with its anchor-free design and decoupled detection head, has become a common baseline in this direction [20,21]. For side-scan sonar images, BES-YOLO [22] enhances YOLOv8 for multi-scale target detection. In addition, Wang et al. [23] proposed a lightweight sonar detector with global feature extraction, utilizing shadow characteristics to improve detection performance while maintaining real-time capability. Nevertheless, existing methods still face two fundamental tensions between practical deployment and accuracy improvement.

First, there is a tension between exploiting cross-modal information and alleviating the intrinsic limitations of sonar imagery. RGB images offer high resolution and rich texture and semantic cues, whereas sonar images suffer from pronounced information deficiency. Although incorporating RGB information can effectively enhance feature representation, RGB and sonar inherently differ in imaging mechanisms, and paired samples across the two modalities are often geometrically inconsistent. As a result, conventional multimodal fusion approaches frequently fail due to the difficulty of reliable alignment.

Second, there is a tension between high-capacity models and resource-constrained platforms. To improve detection accuracy, researchers tend to adopt larger and deeper networks, which inevitably leads to a surge in parameter count and computational cost. For embedded platforms with stringent resource budgets and real-time requirements (e.g., autonomous underwater vehicles), such high-performance detectors are difficult to deploy directly, while overly aggressive model compression typically degrades feature representation and causes a substantial drop in detection accuracy.

To address the above problems, recent studies have begun to focus on knowledge distillation, multi-modal learning, and lightweight design. Knowledge distillation (KD) [24] has been proven to be an effective means of improving the performance of compact models by enabling performance transfer under the teacher–student paradigm; Ghost networks [25] reduce redundant computation via cheap feature generation; cross-modal distillation methods attempt to transfer optical semantics to the sonar domain [26,27,28,29]. Among them, CMOLD [28] achieves object-level cross-modal distillation without requiring strict pixel-wise RGB–sonar alignment, representing a typical approach. We further address practical deployment requirements, emphasize lightweight inference, and adopt a branch-aware heterogeneous distillation mechanism that focuses distillation on more robust semantic branches.

To this end, we propose a novel lightweight cross-modal heterogeneous distillation method (CMHD) for underwater sonar object detection. CMHD addresses two forms of heterogeneity: (1) modality-level heterogeneity: RGB modality vs. sonar modality, and (2) architecture-level heterogeneity: high-capacity teacher YOLOX-M vs. lightweight sonar student network. Under the paired RGB–sonar training setting, CMHD improves sonar detection performance through cross-modal semantic transfer, while retaining only the sonar student network during inference for low-overhead deployment.

The main contributions of this paper are as follows:

- A lightweight underwater sonar object detection method CMHD is proposed. RGB images are innovatively leveraged as semantic support during training to compensate for the information deficiency of the sonar modality, while a distillation paradigm ensures high inference efficiency, providing a lightweight solution that balances effectiveness and accuracy for underwater sensing.
- A branch-aware heterogeneous distillation strategy is designed. To address geometric inconsistencies between RGB and sonar, the distillation constraints are imposed on the objectness and classification branches, whereas bounding-box regression is supervised solely by sonar annotations. This strategy exploits RGB semantic guidance while avoiding unreliable localization signals induced by cross-modal discrepancies.
- A YOLOX-S-Ghost student network with Coordinate Attention (CA) enhancement and a lightweight neck is constructed. CA is incorporated into the YOLOX-S backbone to strengthen spatial position awareness, and the neck is redesigned with Ghost modules. Experimental results show that the student network achieves efficient multi-scale feature fusion under extremely low complexity.

## 2. Our Method

### 2.1. Overall Architecture of the CMHD Network

**Figure 1:**
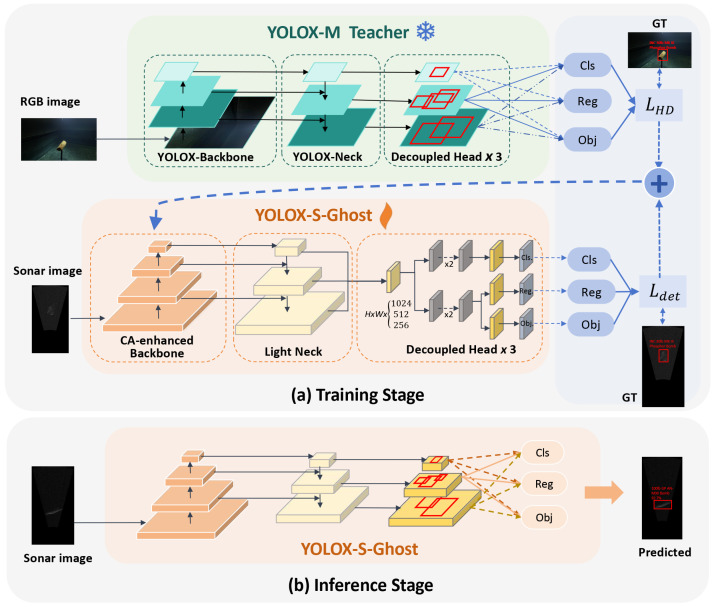
Overall architecture of the proposed CMHD method. It consists of an RGB YOLOX-M teacher network, a lightweight sonar student network, and a cross-modal heterogeneous distillation module. The blue snowflake indicates that the teacher network is frozen, and the orange flame indicates the trainable part during optimization.

Following the YOLOX detection paradigm [9], CMHD adopts a teacher–student training mechanism: the teacher network is a high-capacity RGB detector based on YOLOX-M, and the student network is a lightweight sonar detector improved from YOLOX-S. The overall architecture of the proposed method is illustrated in Figure 1. During the training phase, semantic knowledge is transferred from the RGB teacher network to the sonar student network; during inference, only the sonar student network is retained, thereby satisfying the computational resource constraints of practical deployment.

CMHD is built around three core designs: (1) Cross-modal heterogeneous distillation: The semantic information from the RGB teacher network is used to supervise the sonar student network, alleviating the problem of insufficient semantic information in sonar images. (2) Branch-aware distillation mechanism: Distillation constraints are applied only to the objectness and classification branches, avoiding unreliable regression guidance caused by RGB–sonar geometric inconsistencies. (3) Lightweight student architecture optimization: A CA-enhanced backbone and a Ghost lightweight neck are introduced based on YOLOX-S to achieve a balance between high efficiency and high accuracy.

CMHD is trained in two stages: (1) Teacher network training: The YOLOX-M teacher is independently trained on RGB annotated data to obtain a stable teacher network with strong semantic discriminability. (2) Student network distillation training: After freezing the teacher parameters, paired RGB–sonar samples are fed as input, the teacher outputs soft supervision signals, and the student is jointly optimized with geometrically projected sonar hard supervision and distillation soft supervision.

In the present study, the paired RGB and sonar images are obtained from the same public UXO multimodal acquisition system under a controlled platform, so synchronized RGB–sonar supervision is available for training. Nevertheless, we acknowledge that strictly paired and high-quality RGB–sonar samples may be more difficult to collect in practical deployment scenarios. Therefore, the proposed method is particularly suitable for a training setting where paired multimodal supervision is available offline, while only the sonar sensor is retained during online deployment.

During the inference and deployment phase, CMHD retains only the sonar student network, without relying on RGB input or the teacher branch, thus incurring no additional sensor cost or online distillation overhead. This training–inference decoupling mechanism enables the method to fully exploit cross-modal semantic compensation during training while satisfying the real-time, power, and storage constraints of underwater platforms during deployment.

### 2.2. RGB-Guided Heterogeneous Distillation

#### 2.2.1. Heterogeneous Distillation Formulation

The distillation process in CMHD is built upon a teacher–student method with paired RGB–sonar samples. For each training sample, the teacher network takes an RGB image as input, while the student network takes its paired sonar image. Both networks output detection predictions at three FPN scales, including box regression, objectness, and classification branches. During distillation, the teacher is frozen and provides soft supervision only, whereas only the student branch is updated via backpropagation. To avoid resolution inconsistency between modalities during network input, all RGB and sonar images are resized to the same input resolution of 640×640 before being fed into the teacher and student networks. This unified preprocessing ensures consistent scale normalization during both training and evaluation.

To ensure multi-scale cross-modal supervision, teacher and student predictions are matched at corresponding FPN levels. When spatial resolutions differ between teacher and student feature maps at a given FPN level, teacher predictions are interpolated to the student resolution before loss computation. Therefore, the resolution difference between RGB and sonar data is handled at two levels in this work: unified input resizing before network inference, and interpolation-based prediction alignment at the distillation stage. This design avoids additional errors caused by resolution mismatch and allows distillation to focus on semantic consistency.

Unlike homogeneous distillation, CMHD simultaneously faces modality discrepancy and architectural discrepancy. Therefore, instead of enforcing strict full-branch alignment, CMHD adopts a more robust selective distillation strategy that prioritizes semantically transferable branches across modalities.

#### 2.2.2. Branch-Aware Distillation Strategy

Considering the intrinsic imaging differences between RGB and sonar modalities, as well as potential geometric misalignment between paired samples, CMHD applies distillation only to semantically related branches: (1) objectness branch (obj): probability alignment; (2) classification branch (cls): distribution alignment; and (3) regression branch (bbox): supervised only by geometrically projected sonar ground truth and excluded from distillation.

The key motivation of this branch-aware design is that objectness and classification encode coarse target presence and category-level semantic information (“whether an object exists” and “which category it belongs to”), which are more transferable across modalities. In contrast, box regression is highly sensitive to geometric consistency; direct cross-modal regression distillation may propagate spatially misaligned teacher signals to the student, causing optimization instability or even performance degradation.

To further suppress the effect of low-quality soft labels, CMHD introduces a teacher-confidence gating mechanism. For any location, let $p_{obj}^{t}$ denote the teacher objectness probability, and let $\max_{c}p_{cls}^{t}\left(c\right)$ denote the maximum posterior class probability from the teacher. The gate mask is defined as:

(1) $$M=1\;\left(p_{obj}^{t}\cdot \max_{c}p_{cls}^{t}\left(c\right)>\tau \right),$$

where 1(·) is an indicator function that outputs 1 if the condition holds and 0 otherwise. In this work, the gating threshold is set to τ=0.5, which was selected empirically based on a preliminary sensitivity study under the same experimental setting. Specifically, we compared three candidate values, τ=0.3, 0.5, and 0.7. The corresponding results show that τ=0.5 provides the best trade-off between precision and recall. Mechanistically, a lower threshold tends to retain more low-confidence teacher predictions and may introduce noisy supervision, whereas a higher threshold filters unreliable locations more aggressively but reduces the number of effective distillation samples, making the transferred supervision sparser. Therefore, τ=0.5 is adopted as a relatively robust compromise between suppressing noisy soft labels and preserving sufficient distillation signals. Therefore, a location participates in distillation only when the teacher is simultaneously confident about target presence and category discrimination; otherwise, it is masked out. This simple binary gating effectively filters unreliable soft labels and reduces noisy transfer from background regions, ambiguous boundaries, and uncertain predictions.

#### 2.2.3. Joint Objective Function

CMHD is jointly optimized by two complementary objectives: a supervised detection objective on sonar annotations and a heterogeneous distillation objective from the RGB teacher network. The former ensures task-faithful localization and recognition in the sonar domain, while the latter transfers cross-modal semantic priors to improve student robustness.

The supervised detection objective follows the standard YOLOX multi-task form:

(2) $$L_{\det}=\lambda_{\mathrm{reg}}L_{\mathrm{iou}}+L_{\mathrm{obj}}+L_{\mathrm{cls}},$$

where $\lambda_{\mathrm{reg}}$ is set to 5, $L_{\mathrm{iou}}$ is the box regression loss, and $L_{\mathrm{obj}}$, $L_{\mathrm{cls}}$ are binary cross-entropy losses with logits. The regression branch is always supervised by geometrically projected sonar ground-truth annotations.

Since bounding-box distillation is disabled in our setting, the heterogeneous distillation objective contains only objectness and classification components:

(3) $$L_{\mathrm{hd}}=\lambda_{\mathrm{obj}}L_{\mathrm{hd}}^{obj}+\lambda_{\mathrm{cls}}L_{\mathrm{hd}}^{cls}.$$

Under the same gate mask *M*, the distillation losses are computed as masked averages over all three-scale prediction locations:

(4) $$L_{\mathrm{hd}}^{obj}=\frac{1}{N_{M}+\varepsilon }\sum_{u}M_{u}\;\mathrm{BCE}\;\left(p_{obj,u}^{s},p_{obj,u}^{t}\right),$$

(5) $$L_{\mathrm{hd}}^{cls}=\frac{1}{N_{M}+\varepsilon }\sum_{u}M_{u}\;\mathrm{KL}\;\left(p_{cls,u}^{t}\;∥\;p_{cls,u}^{s}\right),$$

where *u* indexes a prediction location (grid cell) on the multi-scale output maps, and $M_{u}\in \left\{0,1\right\}$ indicates whether location *u* passes the teacher-confidence gate ($M_{u}=1$) or is ignored ($M_{u}=0$). $N_{M}=\sum_{u}M_{u}$ is the number of selected locations used in distillation. $p_{obj,u}^{t}$ and $p_{obj,u}^{s}$ denote the teacher and student objectness probabilities at location *u*, respectively. $p_{cls,u}^{t}$ and $p_{cls,u}^{s}$ denote the teacher and student class posterior distributions at location *u*. BCE(·,·) is the binary cross-entropy for objectness alignment, and KL(·∥·) is the Kullback-Leibler divergence for class-distribution alignment. We set $\varepsilon =10^{-6}$ for numerical stability to avoid division by zero when $N_{M}=0$.

The distillation losses complement the corresponding supervised objectness and classification objectives during optimization. In our experiments, the weights are set to $\lambda_{\mathrm{obj}}=0.5$ and $\lambda_{\mathrm{cls}}=0.1$. This setting keeps sonar hard-label supervision dominant while enabling stable transfer of cross-modal semantic priors from the RGB teacher network, thereby improving the robustness of the lightweight student on low-quality sonar imagery.

Finally, the overall training objective is written as:

(6) $$L_{\mathrm{total}}=L_{\det}+L_{\mathrm{hd}}.$$

This joint formulation balances task-faithful sonar supervision and cross-modal semantic transfer, yielding stable optimization and improved detection robustness under challenging sonar conditions.

### 2.3. Lightweight Student Network Architecture

**Figure 2:**
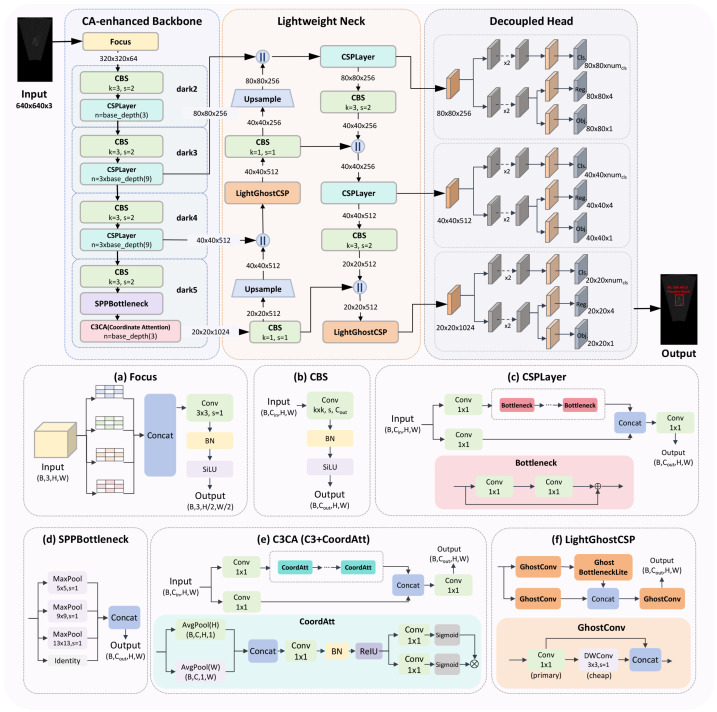
Detailed structure of the YOLOX-S-Ghost detector. The upper panel summarizes the end-to-end data flow from the CA-enhanced backbone through the lightweight neck to the three-scale decoupled detection head, with tensor resolutions annotated at key stages. The lower panels provide block-level definitions used in implementation, serving as a visual specification of the modules instantiated in the student network.

To jointly achieve high detection performance and low deployment overhead in underwater sonar scenarios, we design a lightweight student detector. The architecture follows the standard YOLOX paradigm and preserves the base scaling configuration of YOLOX-S, while introducing sonar-oriented structural enhancements. Specifically, the student network is improved from two perspectives: high-level semantic modeling and multi-scale feature fusion. We introduce CA in the high-level backbone stage and LightGhostCSP modules in key neck fusion layers. The overall architecture is illustrated in Figure 2.

#### 2.3.1. CA-Enhanced Backbone

In sonar images, object boundaries are often weak and noise interference is strong; therefore, relying solely on a conventional convolutional backbone may lead to insufficient high-level semantic discriminability. To address this issue, we introduce CA [30] at the final backbone stage. CA strengthens the coupling between spatial positional information and channel responses through direction-aware context encoding. Compared with the standard high-level backbone path, the enhanced path applies attention-based recalibration after downsampling and SPP-based semantic aggregation, yielding more target-relevant high-level features for the neck.

For an input feature map $X\in R^{C\times H\times W}$, CA aggregates context along the height and width directions and generates directional attention maps. The recalibrated feature is:

(7) $$X_{c,h,w}^{\prime }=X_{c,h,w}\cdot a_{h}\left(c,h\right)\cdot a_{w}\left(c,w\right),$$

where $a_{h}$ and $a_{w}$ denote the height-wise and width-wise attention, respectively. The intermediate channel dimension is set as:

(8) $$C^{\prime }=\max\;\left(8,\left\lfloor \frac{C}{r}\right\rfloor \right),$$

to control additional computation, where *r* is set to 32. This design improves the stability of high-level semantic features without significantly increasing complexity, and is particularly beneficial for weak targets and blurred contours.

#### 2.3.2. Ghost-Based Lightweight Neck

To reduce computational complexity while maintaining feature fusion capability, we adopt a lightweight neck in the student network. Instead of fully replacing the original YOLOX PAFPN with Ghost-style modules, we use a partial replacement strategy. Specifically, LightGhostCSP is introduced at two high-cost fusion nodes: the 40×40 fusion node in the top-down path and the 20×20 fusion node in the bottom-up path. The remaining fusion nodes are kept as standard CSPLayer blocks. This selective replacement reduces parameters and FLOPs while avoiding the representation degradation that may arise from replacing the entire neck, thereby preserving stable multi-scale feature fusion.

As illustrated in Figure 2, LightGhostCSP follows a CSP-style split-transform-merge structure. One branch applies lightweight GhostConv and GhostBottleneckLite transformations, while the other branch provides a lightweight feature path. The two branches are concatenated and further fused to generate the output feature map. Within LightGhostCSP, GhostConv replaces the full convolutional generation of all features with a two-step mechanism of “primary feature generation + cheap feature expansion.” Given an input feature *X*, intrinsic features are first produced by a primary convolution, and complementary features are then generated by a low-cost depthwise operation:

(9) $$X_{p}=f_{\mathrm{pri}}\left(X\right),\;X_{g}=f_{\mathrm{cheap}}\left(X_{p}\right),\;Y=\left[X_{p},\;X_{g}\right].$$

where $f_{\mathrm{pri}}\left(\cdot \right)$ denotes the primary convolutional transform, and $f_{\mathrm{cheap}}\left(\cdot \right)$ denotes the cheap feature generation transform. The concatenated features are then fused and channel-adjusted to obtain the required output dimension for subsequent feature fusion or detection layers.

The cheap feature generation branch is implemented with depthwise convolution to further reduce computation. Overall, this neck design preserves the bidirectional information flow of PAN-FPN while improving the efficiency and practicality of the student network under resource-constrained deployment settings.

## 3. Dataset and Experimental Environment

### 3.1. Dataset

The original UXO dataset [31] comprises 95 recording trajectories and 74,437 sonar frames. Among these, 48,462 frames are paired with camera images, and 37,278 camera frames are provided with bounding-box annotations. The dataset covers three practical UXO categories, and each frame containing exactly one target. Nevertheless, the original sonar images lack bounding-box annotations, and high temporal redundancy between consecutive frames limits their direct utility for supervised learning.

**Figure 3:**
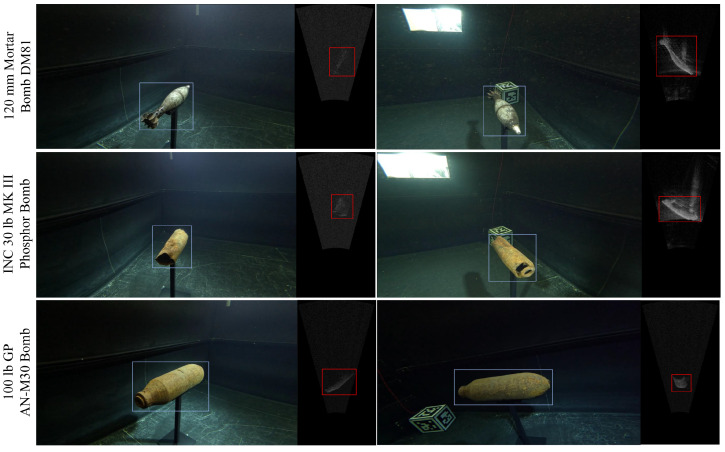
Examples of paired RGB and sonar frames from the UXO^†^ dataset. Blue boxes denote RGB annotations and red boxes denote sonar annotations for the corresponding target in the matched frame pairs.

To address these problems and enhance dataset usability, we constructed the UXO^†^ dataset based on the original release. Using the target’s initial pose, gantry motion trajectories, sonar extrinsics, and ARIS polar imaging geometry from system calibration, we projected the 3D UXO model onto sonar images frame by frame and generated physically grounded axis-aligned bounding boxes. These sonar bounding boxes were generated automatically through geometry-based frame-by-frame projection rather than manually drawn from scratch. To improve annotation quality, we additionally conducted manual visual inspection of the projected results and checked samples with obvious misalignment or anomalies. When necessary, such problematic samples were handled during dataset curation to reduce the influence of possible sensor calibration drift, gantry motion errors, and sonar imaging noise as much as possible. Nevertheless, we note that this physics-guided annotation strategy may still be affected by slight residual calibration or motion errors and therefore is not fully equivalent to exhaustive frame-by-frame manual annotation. Moreover, since the current experimental validation is conducted on the UXO^†^ subset with 1800 paired training samples, the present study should be understood as a focused case-study style verification of cross-modal heterogeneous distillation under this representative RGB–sonar paired setting rather than a broad demonstration of universal generalization. For sampling, we first randomly selected 10 complete capture trajectories with paired RGB bounding-box annotations for each category, then uniformly sampled 100 frames from each trajectory, and finally split the data at the trajectory level into train/val/test with a ratio of 6:2:2. The resulting UXO^†^ dataset contains 1800 paired RGB–sonar image sets for training, 600 for validation, and 600 for testing, with equal class distribution across all splits. Figure 3 shows representative paired RGB and sonar sensor samples.

### 3.2. Experimental Settings

All experiments were conducted in Python 3.8.20 with PyTorch 2.4.1 and CUDA 12.2 on Ubuntu 20.04.5 LTS, using a workstation equipped with an NVIDIA GeForce RTX 4090 GPU and a 13th Gen Intel Core i9-13900K CPU. The input resolution was fixed at 640×640 for all models. During training, all models were optimized for 50 epochs with a batch size of 8. Stochastic gradient descent (SGD) was adopted as the optimizer, with an initial learning rate of $1.25\times 10^{-3}$, a final learning rate of $6.25\times 10^{-5}$, momentum 0.9, and weight decay $5\times 10^{-4}$.

## 4. Experimental Results and Analysis

### 4.1. Ablation Study

To validate the effectiveness of both the proposed student design and teacher selection in CMHD, we conduct four experiments. For the experimental results, all AP and mAP metrics are reported as AP@[0.50:0.95]. Since the adopted subset contains one dominant UXO^†^ instance per image, AR is reported with maxDets = 1. All reported values are expressed in percentages (%).

Student ablation: Fixing the teacher network (YOLOX-M) and comparing different lightweight student models under distillation. As shown in Table 1, when using the same teacher network (YOLOX-M), YOLOX-S-Ghost consistently outperforms the equally lightweight YOLOX-Nano across all three categories in both AP and AR, with relative gains of 14.5% and 13.8% in overall mAP and mAR, respectively. These results indicate that, under lightweight constraints, the proposed student architecture is better optimized for capturing the unique acoustic features of sonar imagery. The significant improvement in the average metrics implies that YOLOX-S-Ghost can effectively mitigate the information loss commonly associated with extreme model compression. This confirms that our design is more receptive to teacher-guided knowledge, thereby serving as a more robust backbone for lightweight underwater target detection.

Teacher ablation: Fixing the student network (YOLOX-S-Ghost) and comparing different teacher models. As shown in Table 2, with YOLOX-S-Ghost fixed, although YOLOX-L and YOLOX-S achieve outstanding performance on certain categories, their performance fluctuates considerably across different categories, particularly underperforming on the more challenging mortar bomb category. In contrast, while YOLOX-M does not achieve the highest scores on every category, it maintains balanced and stable performance across all categories, with a clear advantage on difficult categories, thereby achieving the best overall mAP/mAR. This suggests that, for lightweight student distillation, a medium-capacity teacher provides a more favorable trade-off between representational strength and knowledge transferability.

Distillation source analysis: To clarify whether the performance gain comes from cross-modal supervision or merely from a better training strategy, we additionally compare three distillation settings: (1) self-distillation with an RGB teacher and a sonar student sharing the same lightweight YOLOX-S-Ghost architecture, (2) sonar-only distillation using a stronger sonar YOLOX-M teacher to supervise the YOLOX-S-Ghost sonar student, and (3) the full cross-modal heterogeneous distillation setting of CMHD using an RGB YOLOX-M teacher. As shown in Table 3, the cross-modal RGB-teacher setting achieves the best overall performance, reaching 79.6% mAP and 82.6% mAR, outperforming both self-distillation and sonar-only teacher distillation. This result indicates that the observed gain is mainly attributable to cross-modal semantic transfer from the RGB teacher rather than to standard distillation alone or a generic training enhancement.

Distillation branch ablation: To assess the role of each branch in branch-aware heterogeneous distillation, we individually activate the classification and objectness distillation losses. The results in Table 4 show three clear trends. First, introducing distillation improves overall performance over pure supervised student training, confirming that teacher guidance is beneficial in sonar detection. Second, objectness distillation provides the most reliable overall gain, suggesting that transferring foregroundness priors is particularly important for suppressing background ambiguity in sonar imagery. Third, classification distillation is especially helpful for the hardest category, indicating improved inter-class discrimination for subtle targets. When both branches are enabled, the model achieves the best overall trade-off between precision and recall, demonstrating that the two branches are complementary and that the full branch-aware design provides the most effective knowledge transfer.

**Figure 4:**
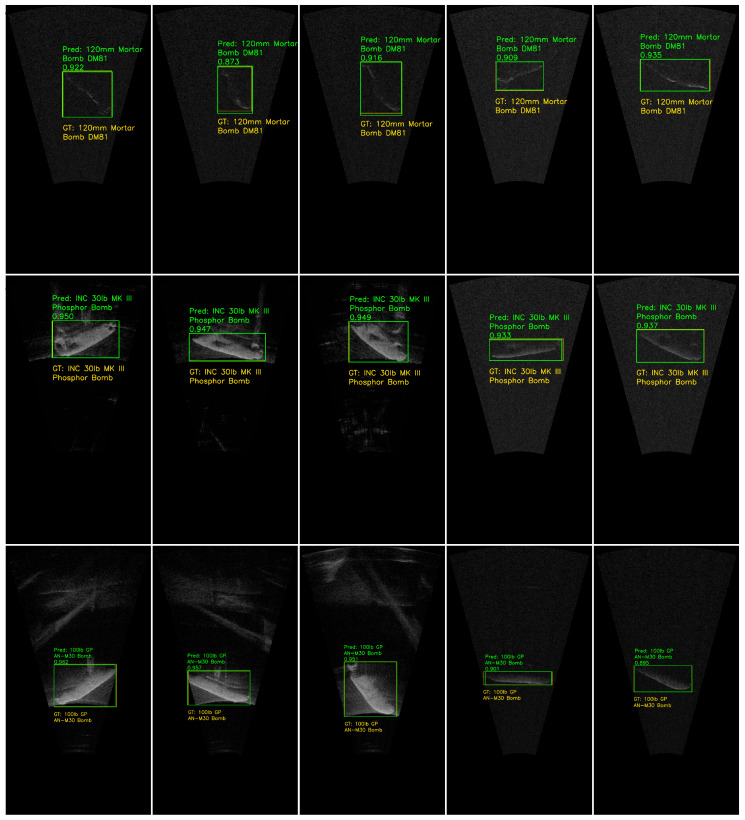
Detection results of CMHD on the test set. Green boxes/labels denote predictions, where “Pred” indicates the predicted category and the number indicates the confidence score. Yellow boxes/labels denote ground-truth annotations (“GT”). The box size and position indicate the target extent and location.

Representative detection examples of the proposed CMHD model are shown in Figure 4, with all samples uniformly selected from the test set to cover diverse scenarios.

### 4.2. Hyperparameter Sensitivity Analysis

To examine the robustness of CMHD to key distillation hyperparameters, we further evaluate the distillation weights $\lambda_{\mathrm{cls}}$ and $\lambda_{\mathrm{obj}}$, and the detailed results are listed in Table 5.

From Table 5, when only classification distillation is considered, $\lambda_{\mathrm{cls}}=0.1$ achieves competitive overall performance, while larger classification weights do not bring further gains and may even reduce mAP/mAR, suggesting that excessive class-level transfer can introduce unstable supervision under noisy sonar conditions. When only objectness distillation is considered, $\lambda_{\mathrm{obj}}=0.5$ performs better than both smaller and larger alternatives, indicating that a moderate objectness weight is more effective for transferring target-presence cues while avoiding over-amplification of noisy responses. When the two branches are combined, the configuration $\lambda_{\mathrm{cls}}=0.1$ and $\lambda_{\mathrm{obj}}=0.5$ achieves the best overall performance, indicating that relatively light classification transfer together with moderate objectness guidance provides the most favorable balance for the current cross-modal sonar detection task.

### 4.3. Comparative Experiment

To evaluate the effectiveness of the proposed CMHD method, we compare it with four representative detectors. All detectors are trained and evaluated on the same geometrically projected sonar labels of the UXO^†^ dataset, with identical input resolution (640×640), 50 training epochs, and the same general optimization settings. The compared methods are configured as follows: (1) YOLOX-Nano [9]. (2) YOLOX-S-Ghost denotes the proposed lightweight student architecture trained without distillation; for a fair architecture-only comparison, both YOLOX-Nano and YOLOX-S-Ghost are trained and tested directly on sonar images. (3) KD-YOLOX-ViT [32] introduces ViT layers into YOLOX and applies single-modal distillation within sonar data by supervising all three decoupled heads (classification, objectness, and regression); in our implementation, YOLOX-M-ViT is used as teacher and YOLOX-S-ViT as student. (4) CMOLD [28] is a homogeneous teacher–student cross-modal distillation method that uses optical images as the teacher modality for sonar student network supervision; similar to CMHD, it distills only classification and objectness branches to avoid unreliable regression supervision caused by RGB–sonar geometric misalignment. Following the homogeneous teacher–student design of the original CMOLD method and the lightweight modeling principle of this paper, our setup uses YOLOX-S-Ghost for both teacher and student architectures. (5) CMHD denotes the complete proposed method, where the YOLOX-S-Ghost is trained with RGB-teacher heterogeneous distillation and only the sonar student network is retained during inference.

As shown in Table 6, CMHD achieves the highest overall performance on the UXO^†^ test set. Compared with the lightweight baseline YOLOX-Nano, CMHD brings significant improvements of more than 12% and 11% in mAP and mAR. This substantial performance leap suggests that, under identical data splits, input resolution, and evaluation protocols, the architectural enhancements and distillation strategy integrated into CMHD jointly produce a strong synergistic effect, rather than merely stacking parameters.

To isolate the distillation effect and validate the standalone performance of the student architecture, we compare YOLOX-Nano and undistilled YOLOX-S-Ghost. Experimental results show that the redesigned YOLOX-S-Ghost outperforms YOLOX-Nano by 14.4% in mAP and 12.7% in mAR. This verifies that the sonar-oriented optimizations for the student model bring prominent performance gains, surpassing conventional lightweight models even without cross-modal knowledge distillation.

To further evaluate the additional benefit of cross-modal heterogeneous distillation, we compare CMHD with YOLOX-S-Ghost, KD-YOLOX-ViT, and CMOLD. Compared with the non-distilled YOLOX-S-Ghost, CMHD yields further gains of 2.6% and 2.7% in mAP and mAR, indicating that distillation effectively bridges the semantic gap that is difficult to overcome with hard labels alone. Compared with KD-YOLOX-ViT and CMOLD, CMHD achieves leading margins of 8.0/6.8 and 4.3/4.0 percentage points, respectively. These comparisons fully demonstrate that, under the current benchmark, the proposed cross-modal heterogeneous distillation mechanism is superior in knowledge transfer efficiency to other distillation or multimodal fusion approaches.

From a category-level view, CMHD achieves outstanding accuracy on the challenging 120 mm Mortar Bomb DM81 class while remaining competitive on the other simpler categories. By contrast, CMOLD gains slight precision on easy classes but degrades severely on hard targets, resulting in imbalanced overall performance. CMHD maintains stable performance across all categories and delivers the best comprehensive metrics. This demonstrates that CMHD avoids overfitting to simple samples; instead, it learns discriminative sonar features to improve robustness against ambiguous and weak-signal targets, achieving more balanced detection.

**Figure 5:**
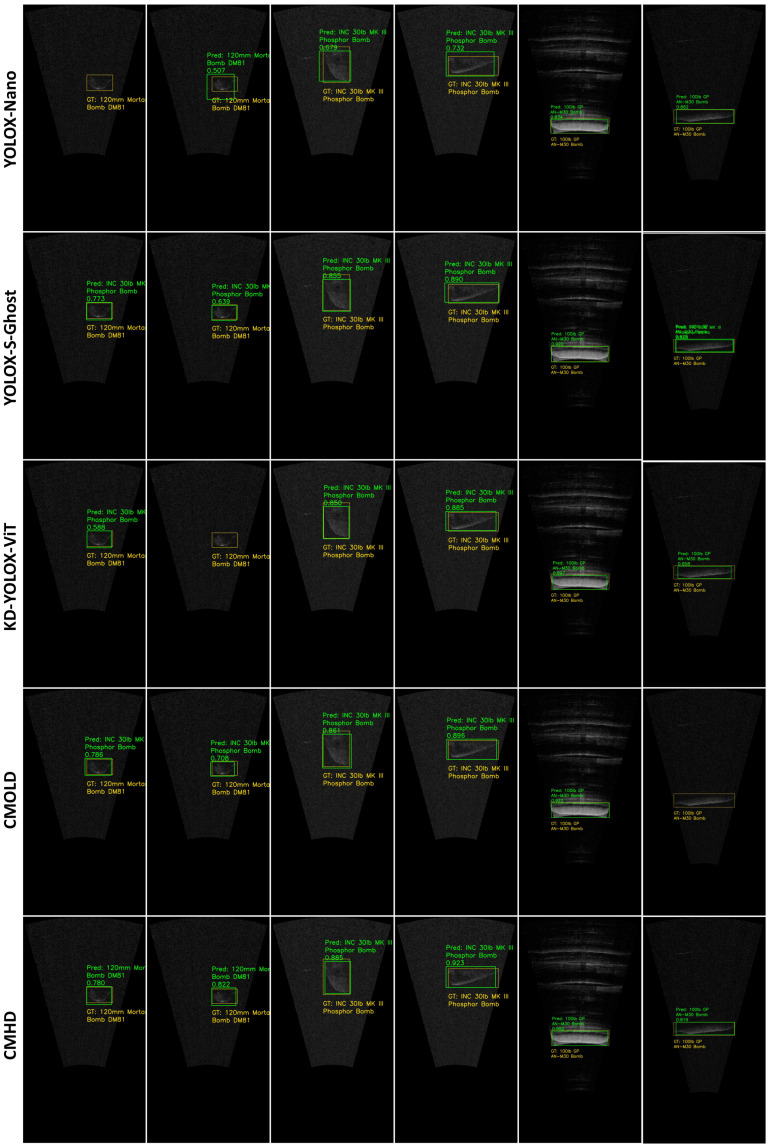
Qualitative comparison of five detectors on six representative UXO^†^ sonar samples. Yellow boxes denote ground-truth annotations, and green boxes denote model predictions with confidence scores.

Figure 5 qualitatively compares five detectors on six typical sonar samples. CMHD achieves more stable detection under complex backgrounds, low-contrast echoes and target variations. It produces bounding boxes with tighter alignment to ground truth, fewer missed targets, and less clutter-caused false alarms on challenging cases. These visual results align well with the quantitative metrics.

**Figure 6:**
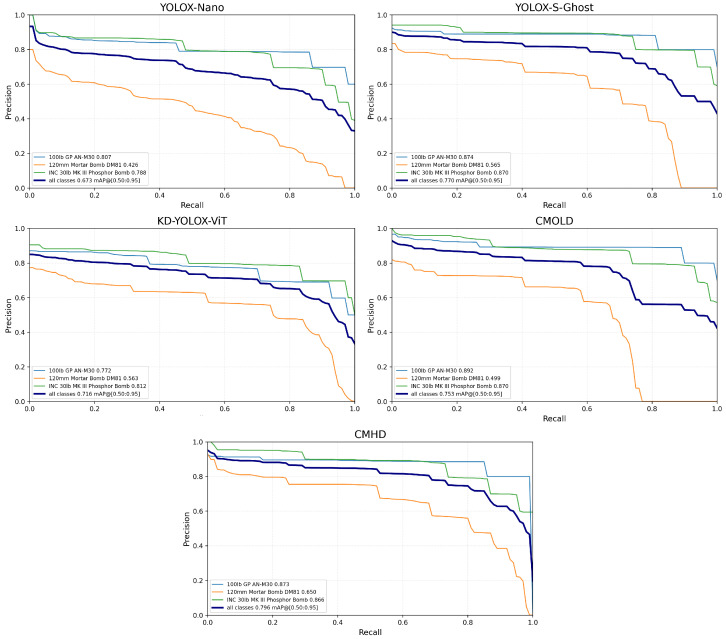
Precision–recall curves on the UXO^†^ sonar test set. Each subplot plots curves under AP@[0.50:0.95], with the legend reporting both per-class and overall mAP results at the same IoU threshold.

Figure 6 shows the precision–recall behavior under AP@[0.50:0.95]. Based on the legend statistics, overall mAP is 0.673 for YOLOX-Nano, 0.770 for YOLOX-S-Ghost, 0.716 for KD-YOLOX-ViT, 0.753 for CMOLD, and 0.796 for CMHD, indicating that CMHD achieves the best overall precision–recall trade-off among the compared methods. At the class level, CMHD achieves AP values of 0.873, 0.650, and 0.866 for the three categories, respectively. The largest advantage is observed on the challenging DM81 class, while performance on the other two classes remains competitive. These trends are consistent with the table-based comparisons.

**Figure 7:**
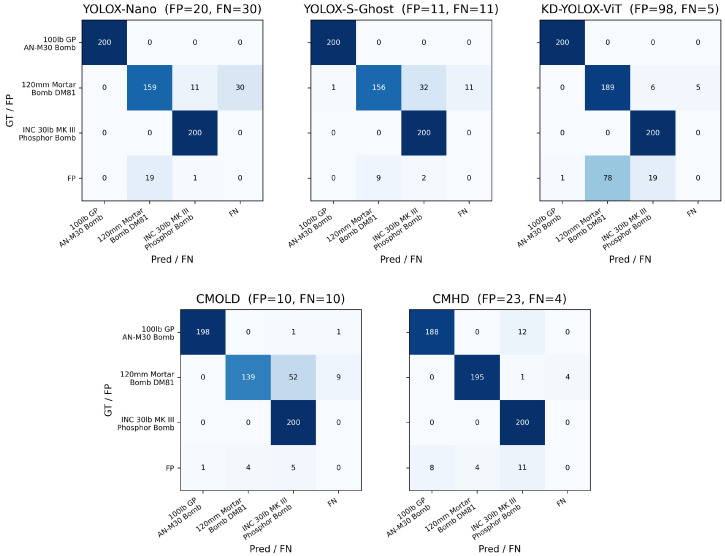
Class-wise confusion matrices of YOLOX-Nano, YOLOX-S-Ghost, KD-YOLOX-ViT, CMOLD, and CMHD on the UXO^†^ test set. The FN column indicates missed detections, and the FP row indicates unmatched false-positive predictions.

To further analyze category-wise behavior, Figure 7 presents the confusion matrices of the five compared methods on the UXO^†^ test set. Because this subset follows a single-object-per-image protocol, two auxiliary bins are introduced: the FN column for missed detections and the FP row for unmatched false-positive predictions.

CMHD exhibits strong class-level consistency, especially on the most challenging 120 mm Mortar Bomb DM81 category, where it achieves the highest diagonal count, surpassing all other models. CMHD also attains the lowest total FN count, indicating the best missed-detection control under the adopted matching rule. For INC 30 lb MK III Phosphor Bomb, all methods obtain 200 correct assignments on the diagonal, suggesting that this category is comparatively easier in the current split.

For 100 lb GP AN-M30 Bomb, CMHD remains highly competitive, with most samples still correctly recognized, while a portion is reassigned to acoustically similar echo patterns in difficult conditions. This trade-off is consistent with the model’s stronger emphasis on hard-category recall and overall robustness. Notably, although CMHD does not yield the smallest FP total, its FP behavior remains controlled and is substantially better than KD-YOLOX-ViT.

### 4.4. Robustness Evaluation on a Challenging Sonar Subset

Because sonar images are often affected by severe speckle noise, blurred contours, and weak edge cues, we further evaluate detector robustness on a manually annotated challenging subset. Specifically, 100 challenging sonar images were selected from the three categories of our dataset, with 33, 33, and 34 images sampled respectively. These images were chosen because they exhibit typical adverse sonar characteristics, including strong speckle noise, weak object boundaries, and low-contrast target responses. All selected images were manually annotated to form a dedicated subset for robustness evaluation.

Quantitative results on the standard test samples and the challenging subset are summarized in Table 7. As expected, all compared methods suffer clear performance degradation under challenging sonar conditions, confirming that heavy speckle noise and weak edge information substantially increase detection difficulty.

Among all methods, CMHD achieves the highest recall on the challenging subset (mAR=54.2%), indicating that it preserves more valid detections under severe sonar degradation. In terms of precision-oriented performance, CMHD reaches an mAP of 46.1%, which remains competitive under the adverse conditions. Although the AP drop from 79.6% to 46.1% is still evident, the results suggest that the proposed method maintains relatively stronger robustness when detection cues become unreliable.

**Figure 8:**
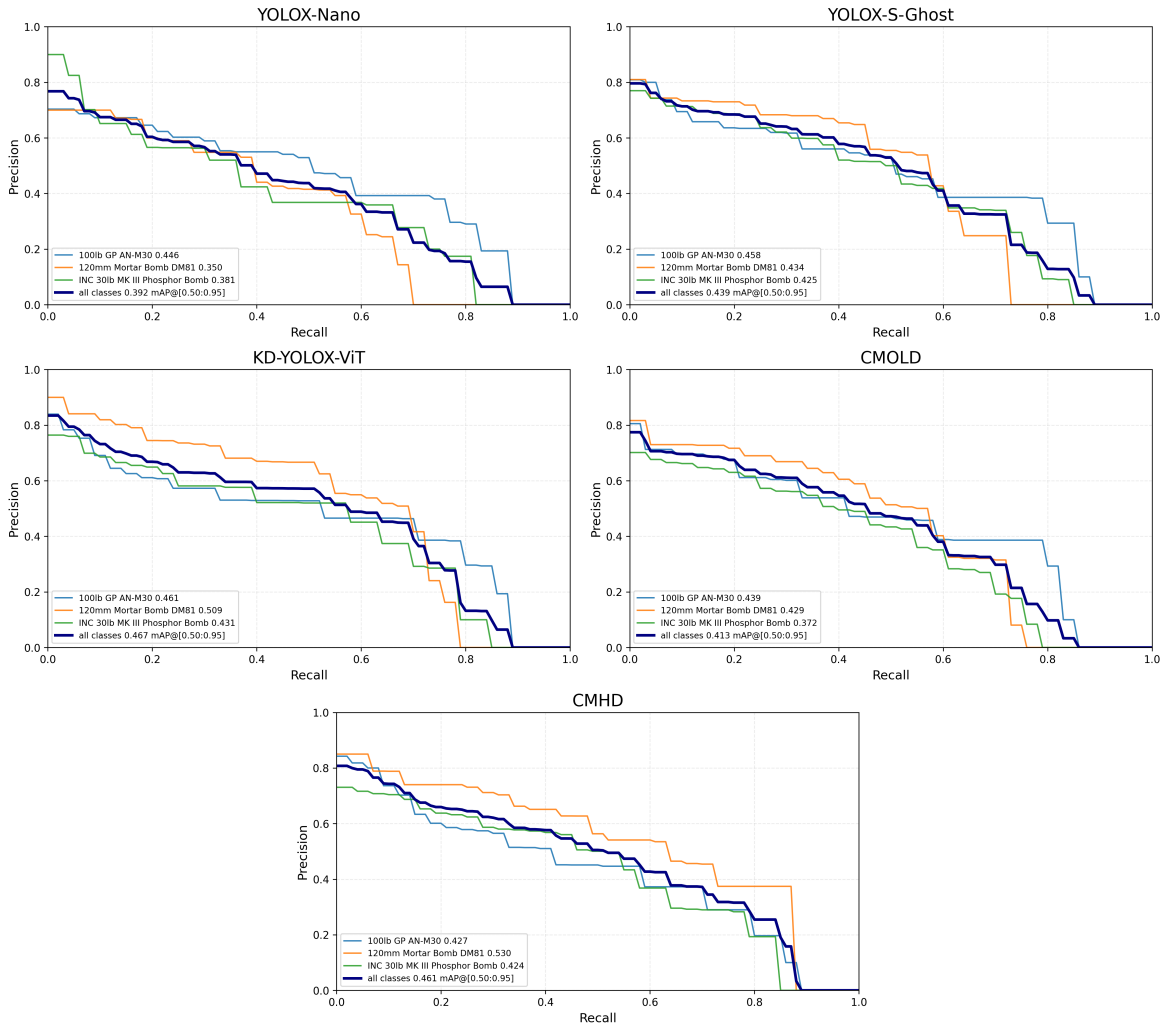
PR curves of different methods on the manually annotated challenging sonar subset.

For a more complete comparison, the PR curves on the challenging subset are shown in Figure 8. The curves further illustrate that all methods experience degraded precision–recall behavior in the presence of severe noise and weak boundaries, while CMHD remains comparatively stable in the high-recall region. This observation is consistent with the quantitative results in Table 7.

### 4.5. Model Complexity and Inference Efficiency Analysis

To further evaluate computational efficiency, model complexity and inference speed are compared between the YOLOX-S and the YOLOX-S-Ghost detector. The YOLOX-S uses the standard configuration [9]. All inference efficiency measurements are conducted on the same RTX 4090 GPU with an input resolution of 640×640 and a batch size of 1. The reported latency corresponds to average per-image inference time (forward + NMS), and FPS is calculated as 1000/Latency(ms). These measurements are intended to compare relative efficiency under a consistent workstation setting, and the results are shown in Table 8.

Compared with the YOLOX-S, YOLOX-S-Ghost achieves a substantial reduction in model complexity: the number of parameters is reduced to approximately one-third, and FLOPs are reduced to less than one-third of the original. In terms of practical inference efficiency, although the latencies of both models are in the millisecond range with only a minor difference, YOLOX-S-Ghost achieves a higher FPS due to its lower computational burden. These results indicate that the proposed student architecture significantly reduces model complexity while maintaining highly competitive real-time inference efficiency. Since CMHD discards the RGB teacher network entirely during inference, its actual deployment complexity is identical to that of YOLOX-S-Ghost.

### 4.6. Failure Case Analysis

**Figure 9:**
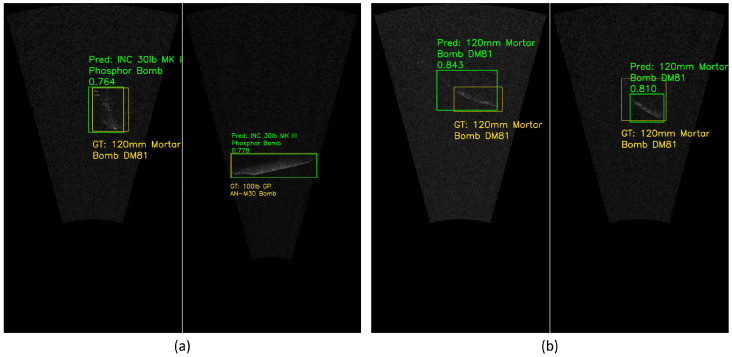
Representative CMHD failure cases on the UXO^†^ sonar test set. Green boxes/labels denote predictions, and yellow boxes/labels denote ground-truth annotations (“GT”). The number after “Pred” indicates the confidence score. Category-label mismatches indicate classification errors, while box mismatches indicate localization inaccuracies. (**a**) Category misclassification cases. (**b**) Localization inaccuracy cases.

Although CMHD achieves the best overall performance, challenging failure cases still occur. Figure 9 presents representative examples from the UXO^†^ sonar test set. Sample group (a) exhibits obvious inter-class confusion; although the category prediction of sample group (b) is correct, the predicted bounding box deviates significantly from the ground-truth target region.

A plausible reason is that sonar returns from different UXO^†^ categories can share similar local echo patterns under certain viewpoints, range conditions, and speckle noise. When class-discriminative cues are weak, the detector may still localize salient regions but assign them to a visually similar class with high confidence. Moreover, blurred contours, low structural detail, and background speckle can also degrade localization accuracy, making the predicted box sensitive to small shape and pose changes, thereby reducing the robustness of IoU-based matching.

These observations suggest that future improvements should jointly address both inter-class ambiguity and localization robustness in sonar imagery, for example via stronger class-discriminative representation learning, confusion-aware hard sample mining, and geometry-aware or shape-aware box refinement strategies under acoustically similar responses.

In addition, because the sonar annotations are generated through geometry-based projection and subsequent manual inspection rather than exhaustive manual frame-by-frame labeling, future dataset refinement should further improve robustness against residual calibration drift, gantry motion perturbation, and sonar-specific imaging noise.

## 5. Conclusions

This paper proposed a lightweight underwater sonar object detection method CMHD, which relieved the contradiction between the inherent semantic scarcity of sonar sensing data and the deployment constraints in real scenarios simultaneously. CMHD adopted YOLOX-M as RGB teacher network and enhanced YOLOX-S-Ghost as sonar student network. Ablation results showed that each component contributed to performance: the YOLOX-S-Ghost student was more effective under lightweight constraints, YOLOX-M provided the best transfer outcome among tested teachers, and branch-level analysis indicated that objectness distillation delivered the most consistent overall gains while combining objectness and classification achieved the best precision–recall balance. Comparative results further demonstrated that CMHD achieved overall performance superior to several representative methods, while exhibiting more stable detection behavior under low model complexity, validating the effectiveness of cross-modal heterogeneous distillation and the proposed lightweight architectural improvements.

In the future, additional validation is required on in situ data that are closer to practical deployment, particularly under more challenging conditions involving stronger noise, larger appearance variations, and denser target layouts, in order to comprehensively assess the generalization ability and stability of the proposed method. Although paired RGB–sonar data can be obtained on controlled multimodal acquisition platforms, such strictly paired supervision is often more difficult to collect in realistic deployment environments. Therefore, to reduce the requirements for data acquisition and annotation, we will investigate ways to decrease the reliance on paired multimodal supervision, for example by leveraging weak supervision, semi-supervised or self-supervised learning, and domain adaptation, so that effective cross-modal knowledge transfer and improved sonar detection performance can still be achieved in the absence of strictly paired RGB–sonar samples.

## Figures and Tables

**Table 1:** Ablation on student network (teacher network fixed as YOLOX-M). Bold values indicate the best performance in each column.

Student	100 lb GP AN-M30			120 mm Mortar Bomb DM81			INC 30 lb MK III Phosphor Bomb		mAP	mAR
	AP	AR		AP	AR		AP	AR		
YOLOX-S-Ghost	**87.34**	**89.75**		**64.99**	**69.70**		**86.64**	**88.25**	**79.6**	**82.6**
YOLOX-Nano	83.77	86.30		44.27	48.40		80.52	83.15	69.5	72.6

**Table 2:** Ablation on teacher network (student network fixed as YOLOX-S-Ghost). Bold values indicate the best performance in each column.

Teacher	100 lb GP AN-M30			120 mm Mortar Bomb DM81			INC 30 lb MK III Phosphor Bomb		mAP	mAR
	AP	AR		AP	AR		AP	AR		
YOLOX-L	88.78	91.40		59.46	62.75		**89.77**	**91.45**	79.3	81.9
YOLOX-M	87.34	89.75		**64.99**	**69.70**		86.64	88.25	**79.6**	**82.6**
YOLOX-S	**90.27**	**91.95**		59.19	64.50		86.12	88.15	78.5	81.5

**Table 3:** Comparison of different distillation sources. Bold values indicate the best performance in each column.

Teacher Model	Teacher Modality	Student Model	Student Modality	100 lb GP AN-M30 Bomb			120 mm Mortar Bomb DM81			INC 30 lb MK III Phosphor Bomb		mAP	mAR
				AP	AR		AP	AR		AP	AR		
YOLOX-S-Ghost	RGB	YOLOX-S-Ghost	sonar	84.8	87.0		62.9	67.2		84.5	87.3	77.4	80.5
YOLOX-M	sonar	YOLOX-S-Ghost	sonar	82.5	86.1		58.7	65.0		85.2	89.1	75.5	80.0
YOLOX-M	RGB	YOLOX-S-Ghost	sonar	**87.4**	**89.8**		**65.0**	**69.7**		**86.4**	**88.3**	**79.6**	**82.6**

**Table 4:** Ablation on distillation branches. Bold values highlight the better-performing results. The symbols *√* and × indicate that the corresponding distillation branch is enabled and disabled, respectively.

	Distillation			100 lb GP AN-M30			120 mm Mortar Bomb DM81			INC 30 lb MK III Phosphor Bomb		mAP	mAR
	cls	obj		AP	AR		AP	AR		AP	AR		
	×	×		87.3	89.6		56.6	61.4		87.2	88.9	77.0	79.9
	*√*	×		84.1	87.5		63.6	69.2		**88.3**	**90.4**	78.7	82.3
	×	*√*		87.3	**90.1**		63.9	67.2		88.1	90.1	**79.8**	82.5
	*√*	*√*		**87.3**	89.8		**65.0**	**69.7**		86.6	88.3	79.6	**82.6**

**Table 5:** Sensitivity analysis of distillation weights. Bold values indicate the best performance within each comparison group.

$\lambda_{\mathrm{cls}}$	$\lambda_{\mathrm{obj}}$	100 lb GP AN-M30 Bomb			120 mm Mortar Bomb DM81			INC 30 lb MK III Phosphor Bomb		mAP	mAR
		AP	AR		AP	AR		AP	AR		
0.1	–	84.1	87.5		63.6	69.2		88.3	90.4	78.7	**82.3**
0.2	–	87.3	89.6		59.4	62.4		85.8	87.9	77.5	80.0
0.3	–	90.1	92.2		59.5	64.9		86.7	89.0	**78.8**	82.1
0.5	–	81.4	84.3		61.9	65.6		86.5	88.6	76.6	79.5
–	0.3	89.1	92.0		55.8	61.1		86.6	89.0	77.2	80.7
–	0.5	87.3	90.1		63.9	67.2		88.1	90.1	**79.8**	**82.5**
–	1.0	86.9	89.1		54.3	59.4		85.2	87.3	75.5	78.6
0.15	0.25	86.2	88.8		63.2	67.9		87.1	89.0	78.8	81.9
0.3	0.5	77.7	81.1		64.3	69.2		86.9	89.8	76.3	80.1
0.1	0.5	87.3	89.8		65.0	69.7		86.6	88.3	**79.6**	**82.6**

**Table 6:** Performance comparison on the UXO^†^ dataset. All AP/mAP values are AP@[0.50:0.95] (%), and all AR/mAR values are AR (maxDets=1) (%). Bold values indicate the best performance in each column.

Method	100 lb GP AN-M30 Bomb			120 mm Mortar Bomb DM81			INC 30 lb MK III Phosphor Bomb		mAP	mAR
	AP	AR		AP	AR		AP	AR		
YOLOX-Nano	80.7	83.5		42.6	48.0		78.8	81.4	67.3	70.9
YOLOX-S-Ghost	87.4	89.6		56.5	61.4		87.0	88.9	77.0	79.9
KD-YOLOX-ViT	77.2	79.9		56.3	64.5		81.2	83.2	71.6	75.8
CMOLD	**89.2**	**91.5**		49.9	54.9		**87.0**	**89.4**	75.3	78.6
CMHD	87.3	89.8		**65.0**	**69.7**		86.6	88.3	**79.6**	**82.6**

**Table 7:** Performance comparison on standard test samples and the manually annotated challenging sonar subset. Bold values highlight the best performance results.

Method	Standard Test Samples			Challenging Subset		AP Drop	AR Drop
	mAP	mAR		mAP	mAR		
YOLOX-Nano	67.3	70.9		39.2	47.0	28.1	23.9
YOLOX-Ghost	77.0	79.9		43.9	50.9	33.1	29.0
KD-YOLOX-ViT	71.6	75.8		**46.7**	53.8	24.9	22.0
CMOLD	75.3	78.6		41.3	51.9	34.0	26.7
CMHD (ours)	**79.6**	**82.6**		46.1	**54.2**	33.5	28.4

**Table 8:** Model complexity and inference efficiency comparison.

Model	Params (M)	FLOPs (G)	Latency (ms/image)	FPS
YOLOX-S	8.94	26.76	3.74	267.38
YOLOX-S-Ghost	3.00	8.31	3.57	280.11

## Data Availability

The public UXO multi-modal dataset used in this study is available at https://github.com/dfki-ric/uxo-dataset2024 (accessed on 16 June 2026). The geometrically projected sonar annotations generated in this study are available from the corresponding author upon reasonable request.
